# Catabolic and anabolic faces of insulin resistance and their disorders: a new insight into circadian control of metabolic disorders leading to diabetes

**DOI:** 10.4155/fsoa-2017-0015

**Published:** 2017-06-26

**Authors:** Polina M Schwartsburd

**Affiliations:** 1 Institute of Theoretical & Experimental Biophysics, Russian Academy of Sciences, Pushchino, Moscow Region, 142290, Russia

**Keywords:** circadian disorders, inflammation, insulin resistance, obesity, metabolism, Type 2 diabetes

## Abstract

Maintenance of glucose homeostasis during circadian behavioral cycles is critical. The processes controlling the switch between predominant lipolysis/fatty oxidation during fasting and predominant lipid storage/glucose oxidation following feeding are determined principally by insulin. Chronic elevated threshold of insulin resistance (IR) is a key pathological feature of obesity, Type 2 diabetes, sepsis and cancer cachexia; however, a temporal reduced threshold of IR is widely met in fasting/hibernation, pregnancy, antibacterial immunity, exercise and stress. Paradoxically, some of these cases are associated with catabolic metabolism, whereas others are related to anabolic pathways. This article considers the possible causes of circadian disorders in glucose and lipid metabolism that act as a driving force for obesity-promoted development of Type 2 diabetes. This is intended to provide improved insight into the pathogenesis of chronic circadian disorders that increase the risk of diabetes, and consider new targets for its metabolic and drug correction.

## Lay abstract

Insulin resistance (IR) is a common adaptive mechanism, acting under opposite anabolic and catabolic conditions. However, chronic IR is a key pathological feature of obesity, Type 2 diabetes and cancer cachexia, whereas a temporal IR is widely seen in fasting, pregnancy, exercise and stress. Therefore, it is important to understand when this transient IR-mechanism shifts to chronic IR-associated diseases. What factors result in the switch between the anabolic and catabolic conditions and what defect(s) in this switch is associated with chronic IR induction? The present opinion article aimed to address these questions to the metabolic changes typical for circadian regulation in lean, obese and diabetic patients.

**Graphical abstract:** Early circadian IR disorders caused by overweight and obesity are associated with increased risk for diabetes via formation of a vicious cycle between lipid anabolic and catabolic programs thus distorting insulin and lipid levels in day/night period.

## Graphical abstract

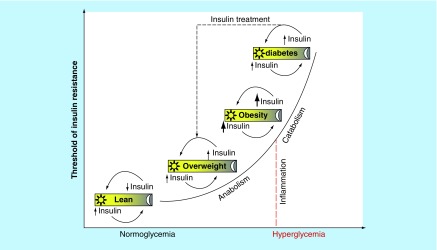

Metabolism is a combination of two processes: anabolism and catabolism. Anabolic pathways require energy to generate macromolecules such as lipid and nucleotides, whereas the catabolic pathway breaks molecules to produce energy. Catabolic and anabolic pathways are interdependent, for example, in the circadian cycles (namely: rest/activity, fasting/feeding, sleep/wake), during which homeostasis is maintained by reciprocal activation of glucose and lipid metabolism [1]. Glucose metabolism is controlled by glucose uptake in adipocyte and muscle cells and inhibits glucose production in liver, thus serving as the primary regulator of blood glucose concentration. Inability of insulin to promote normal glucose uptake by fat and muscle, and inhibit hepatic glucose production (termed insulin resistance [IR]) can be observed widely in physiology and pathology [2]. Indeed, chronic IR is a key feature of Type 2 diabetes and cancer [3], and its predecessors such as obesity and chronic low-grade inflammation; whereas transient IR also occurs as an adaptive response to circadian fasting, hibernation and pregnancy. Little is known about why IR occurs in so many functional states and what controls the analyzed IR shifts from the physiological to pathological states. What are the reasons for the induction of different states that is associated with IR at wide-ranging glucose levels and its dependence on activation of catabolic or anabolic programs? An answer to these questions might be achieved through the hypothesis [4], in which IR is considered as a two-sided mechanism acting under opposite catabolic and anabolic conditions. It is reasonable that such dualism helps to sustain glucose homeostasis in circadian metabolism, namely, in healthy lean individuals, or gains circadian disorders in overweight, obesity and diabetes patients. To test this hypothesis, I performed a comparative ana­lysis of different cases of circadian transfers from daily anabolic to night catabolic programs and its IR disorders that are typical of lean, overweight and obese patients. The main objective of this study was to investigate the possible cause(s) of the circadian disorders in glucose and lipid metabolism that acts as a driving force for obesity-promoted development of Type 2 diabetes. The present viewpoint is also focused on the ana­lysis of implication of catabolic and anabolic pathways in the development of the pathological form of IR for revealing the new targets for its correction in the analyzed metabolic disorders.

## Insulin resistance & circadian metabolism: comparison of reciprocal glucose-lipid regulation between lean, overfeeding, obesity & diabetes

The survival of multicellular organisms depends on the organism's ability to maintain glucose homeostasis in times of low/high nutrient availability or low/high energy needs. These effects are achieved by the organism's ability to support equilibrium between energy-producing catabolic processes and energy-consuming anabolic pathways that make possible support of the metabolic homeostasis in fasting/feeding and sleep/wake cycles. Glucose metabolism is subject to fundamental systemic regulation that is controlled by the anabolic hormone insulin. In mammals, insulin is produced by pancreatic beta cells and released into the blood stream in response to increased concentrations of glucose. Insulin increases glucose uptake in the insulin-sensitive tissue (such as muscle and fat ones) and inhibits hepatic glucose production [5], thus serving as the primary regulator of blood glucose concentration in a narrow range between 4 and 7 mM in normal individuals [6]. Insulin increases the glucose uptake in cells by stimulating the translocation of the glucose transporter GLUT4 from intracellular sites to the cell surface. Insulin stimulates the glucose uptake primarily in muscles because up to 80% insulin-dependent glucose disposal occurs in activated skeletal muscles that require effective glucose delivery for its high-energy demand; whereas free fatty acids (FFA), especially saturated fatty acids (SFA) reduce the insulin-mediated glucose uptake in adipocytes and skeletal muscles [7]. Because of this, the increase in FFA induces IR that is contributing to supported glucose levels by its deficit. However, a similar IR response can occur at an increased glucose level. These results raise questions about a possible organization of IR as a two-side mechanism supporting glucose homeostasis by low/excess availability of glucose [4]. How can this mechanism be acted by the circadian clock in going from rest/fasting to active/feeding phases?

**Figure 1.**
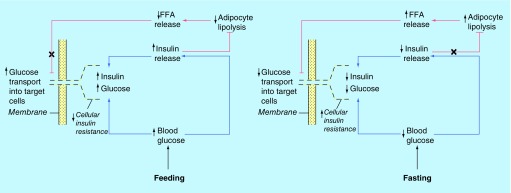
**Dual signal model of reverse day/night switching between lipid and glucose metabolism involved in adaptive induction of insulin resistance-response.** When operated at a glucose-transporting pathway the shutdown of lipid-transporting pathway is observed under catabolic night/fasting conditions. Such an effect is attended with insulin-controlled inhibition of adaptive lipolysis and reduction of circulating FFAs in the active day period. In contrast, when the insulin concentration returns to its normal low level as in night, the inhibiting effect of insulin on fat lipolysis is abolished and the level of circulating FFAs is growing in complexity with the shutdown of glucose transport into insulin-controlled cells (named insulin resistance). FFA: Free fatty acids.

**Figure 2.**
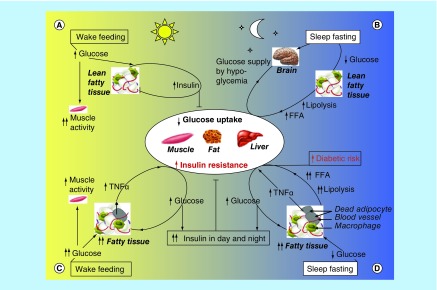
**Day/night balance point between the two antagonistic anabolic and catabolic programs could provide a sensitive on/off switch between glucose and lipid metabolism in healthy lean (not overweight) humans.** Balanced **(A & B)** and unbalanced **(C & D)** feedback regulation of glucose-lipid metabolic switching between daily feeding and sleep fasting states typical of healthy lean **(A & B)** and overweight/obese **(C & D)** individuals is presented. The capacity of metabolic switching between glucose and fat fuel is a key feature of healthy individuals **(A & B)** that are characterized by an increase in glucose and insulin metabolism in day period and its decrease at night fasting. In overweight/obesity-induced insulin resistance, these natural day–night metabolic responses break down and become pathology through the induction of hyperinsulinemia, hyperlipidemia and chronic insulin resistance in the whole period of the day–night cycle **(C & D).**

In mammals, the clock system maintains homeostasis of behavioral and energetic states during alternating phases in sleep/wake and fasting/feeding cycles [8,9]. This is achieved by the use of metabolic switch between catabolic and anabolic programs relating to glucose and lipid metabolism when needed. For example, if organism is unable to take up enough ‘fuel’ for the maintenance of metabolic homeostasis, it would be forced to activate the catabolic program and switch from glucose to lipid metabolism in parallel with restriction of insulin secretion and glucose uptake into adipocyte and muscle cells that acquired IR (both effects are seen during sleep/fasting [8,9] and hibernation [10]). In human, during the daytime there is a peak of insulin secretion to increase energy utilization and storage, because insulin can act as one of the key feedback regulators that the circadian switch from glucose to lipid metabolism in a reciprocal manner [8]. A simplified two-cycle model for the glucose-lipid switch by insulin-controlled feedback regulation is shown in Figure 1. The proposed model illustrates that when the level of glucose is elevated after a daily meal, insulin works to deliver a greater part of blood glucose into the operating skeletal muscles (Figures 1 & 2A), in parallel with inhibition of fat lipolysis in adipocytes which reduces the concentration of circulating lipid and FFA. As the levels of insulin and blood glucose are reduced in the sleep/fasting state (Figures 1 & 2B), the insulin-mediated suppression of fat lipolysis is cancelled. Therefore the concentration of circulating FFA increases and lipid becomes an important energy source in the circadian rest phase. From this is follows that the dual function of the white adipose tissue to switch on/off between lipolysis and lipogenesis [9] together with transient FFA-induced IR responsible for glucose redistribution [10] can act as a key regulator of the circadian glucose homeostasis. It is also important to note that FFA-induced IR takes place in resting skeletal muscles requiring no glucose consumption during sleep. This reserved glucose then will be available to glucose-sensitive organs such as the brain. This adaptive mechanism could compensate temporarily for glucose deficiency in neurons, usually consuming 70% of glucose and oxygen supplied to the brain [11]. In other words, the ability of FFA to induce the adaptive IR may be part of the coordinated response that could protect glucose-sensitive cells against hypoglycemia developing during physiological circadian starvation (such as human night/fasting or animal hibernation). Under these conditions, IR could have no negative consequences for skeletal muscles, because they are in the resting inactive state during sleep. Why can the opposite IR effect be expected in adipose tissues by a shift from leanness to overweight, obesity and diabetes that maintain chronic IR in day and night periods, in contrast to healthy normal weight? What is the metabolic basis of circadian disorders by high fat diet and obesity?

The current mode of life with high fat-glucose diet, low physical activity, shift work and chronic stress is followed by increased fat storage, systemic IR and substantial elevation in serum insulin, triglyceride, FFA levels that enhances the risk of circadian disruption [12,13]. The day-night impaired switch between the glucose and lipid uptake as the fuel is based on mitochondrial incomplete fatty acid oxidation in day and night periods seen in parallel with retention of hyperinsulinemia, hyperlipidemia and increased blood FFA [8,14,15,8]. Among FFA only SFA, but not unsaturated fatty acids, usually correlate with the IR effect [15]. In such a manner lipolysis-induced SFA can lead to an elevated threshold of cellular unresponsiveness to insulin, as exemplified by the fasting state during sleep. However this relationship is also preserved in overweight individuals in day–night time, as the SFA level increases. A reciprocal relationship between SFA-induced IR in fat/muscle cells and circulating insulin is observed, because the daily elevated insulin concentration is able of compensating the night-induced IR in healthy lean individuals. Figures 2 illustrates what is known for the feedback regulation between glucose, insulin and lipid metabolisms and their key interconnections by the wake-sleep circadian cycle in normal lean (Figure 2A & B) and overweight (Figure 2C & D) individuals. According to this model, the day elevated level of insulin in healthy lean individuals in day (Figure 2A) acts as one of the key feedback regulators that is able to restore the night increased threshold of insulin action (Figure 2B). This reversible circadian effect is often missing in overweight individuals who retain IR in wake (Figure 2C) and sleep period (Figure 2D), possibly via maintenance of a relatively elevated FFA/SFA and insulin leading to loss of circadian rhythm [15,16] Such metabolic abnormalities occur before development of diabetes but this prepathological state can be improved through diets, containing low levels of lipids enriched with SFA, and/or corrective action of metformin and resveratrol [17,18].

Is fat weight gain pathology or not? Although the exact role of IR under excess nutrients is unclear, IR may function as both a sensor of nutrient stores and as an instructive signal for tissues to switch from glucose to fatty acid metabolism [12]. It was found earlier that the number of fat cells (adipocytes) is constant during human life [19]. Overfeeding leads to stimulation of lipogenesis and accumulation of intracellular fat that raises its volume and formation of hypertrophic adipocytes. As a result, the ratio of the insulin receptor number to the increased size of the adipocyte surface is decreased, causing an increment of the IR-threshold in high-fat hypertrophic adipocytes, probably for its protection against lipid over-storage [6] and oxidative damage [20]. Under these conditions, adipocytes tend to hypertrophy and chronic hypoxia that give an increased risk of fat cell death; their breakdown products can act as local catabolic signals for recruitment and activation of macrophages and B2 lymphocytes [21,22]. The proinflammatory released cytokines, such as TNF-α and IL-6, activate lipolysis. Moreover, these cytokines and leukotriene B4 can act in a paracrine manner to activate the intracellular proinflammatory pathways in neighboring cells [21,22] and in an endocrine manner leading to inflammation in distal tissues (such as liver and skeletal muscles).

Elevated levels of proinflammatory cytokines can also contribute to IR by antagonizing insulin signaling, thereby inhibiting insulin-dependent glucose uptake. Mice with impaired insulin sensitivity in response to TNF gain the capacity to be protected against obesity. This is achieved by TNF blockades through genetic removal of the TNF/TNF-receptor [23] or immunological neutralization of TNF [24]. As a result, it was concluded that obesity-associated low-grade inflammation and lipolysis-elevated FFA can be contributed to the etiology of inflammatory-supported IR disrupting circadian clock stimulating the advent of Type 2 diabetes [25].

Overweight and obese people get low-grade inflammation and IR, the induction of which closely correlates with increased production of insulin and FFA. The FFA derived from high-fat adipocytes has been suggested to contribute to IR by inhibiting the glucose uptake and its oxidation, glycogen synthesis and incomplete β-oxidation of fatty acids [26–28]. A combination of these metabolic changes occurs in obese people. It may be a result of the deregulated feedback cycle between the up-growing FFA and inadequate increment of insulin production. At the initial period these feedback tend to increase the insulin level that is able to compensate for the FFA-increased threshold of glucose uptake into hypertrophic adipocytes thus maintaining a normal level of blood glucose. However the next period of chronic obesity may shift to diabetes by creating a vicious cycle between ahead growing FFAs (as in day [Figure 2C] and night [Figure 2D] periods) and insufficient increment of insulin production, which cannot compensate for the increased IR-threshold [14,15]. As a result, the blood glucose concentration is elevated with retention of increased blood FFA and insulin levels in wake and sleep periods. Together, these circadian disorders can be considered as a pathological basis for further development of prediabetes state, which requires correction.

**Figure 3.**
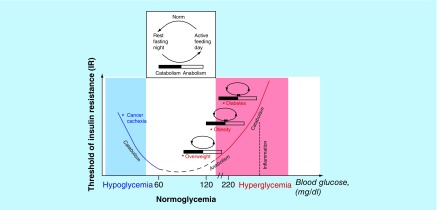
**Schematic illustration of the coupling of blood glucose level, insulin resistance and circadian phase shift in the transition from normal weight to overweight, obesity and diabetes that may be approximated by the U-like dose-response curve model.** The circadian system is regulated by the balance between energy-producing catabolic processes and energy-consuming anabolic process, and its metabolic switch from one process to another. It controls lean individuals (white color) in the day period of wakefulness and feeding coincides with activated metabolism and thermogenic state, whereas sleep and fasting correspond to reduced metabolism and activated catabolism. This circadian control between predominant lipolysis/fatty acid oxidation during circadian fasting and predominant lipid storage/glucose oxidation following feeding is determined principally by insulin and adaptive insulin resistance (IR). The transition from leanness to overweight and obesity can disrupt the normal circadian rhythm, in parallel with chronic activation of catabolic programs (inflammation, lipolysis and stress), mediators of which such as inflammatory cytokines, saturated fatty acids and glucocorticoid share the common property – induction of IR supporting the circadian disorders in the adipose tissue (the effect is illustrated as a right and downward shift from the white to red color region on the U-curve). Transition to diabetes correlates with the formation of vicious cycles maintaining chronic IR and circadian disorders: as the adipocyte tissue becomes unresponsive to insulin the pancreas compensates by secreting ever more anabolic insulin, and gradually tissues grow a higher threshold of resistance to insulin action. It can be assumed that concurrent interactions between both activated catabolic and anabolic programs alter circadian glucose-lipid rhythms and support the chronic inflammatory-mediated IR state in the adipose tissue, which can form a pathological basis for development of diabetes (red color region).

However extreme diet-induced obesity, accompanied by systemic IR, hyperinsulinemia, and hyperlipidemia, is observed as an evolutionary conserved, adaptive and entirely pathology-free response in obese hibernators [10]. It is important that these obese hibernators demonstrate no pathological consequences of their brief bout with obesity, simultaneously failing to develop inflammation and insulin in adipocytes by hibernation. It is reasonable to suggest that obesity-induced low-grade inflammation, chronic lipolysis, increased FFA and insulin levels are a driving force responsible for the key pathogenic shift from lipogenesis to local activation of the catabolic program, in part lipolysis-stimulated IR and hepatic gluconeogenesis, promoting hyperglycemia through increased glycerol and FFA delivery to the liver [25]. Earlier it was found [26] that circadian-induced gluconeogenesis contributes to 50% of glucose production after an overnight fast in healthy humans, whereas obesity-induced humans is attended with circadian metabolic imbalance, namely the increased release of glucose from the liver and its reduced uptake from peripheral tissues in day/night period. These changes are observed not only in night fasting but also in day feeding periods [27]. It is known that IR and gluconeogenesis are usually induced in night catabolic condition with aim to protect organism against fasting-stimulated hypoglycemia. However the unexpected activation of gluconeogenesis in anabolic condition can lead to persistent hyperglycemia that is a major contributing factor to diabetes development via circadian metabolic disorders [27]. A simple model of this pathological circadian state is shown in Figure 3. The model indicates when the catabolic shift may arise by anabolic condition (excess nutrients: glucose, FFA, glycerol) that leads to chronic IR and prediabetic hyperglycemia (Figure 3, right side). This shift is based on chronic permanent production of inflammatory cytokines and FFA/SFA providing a feed-forward loop for the maintenance of vicious lipolysis and inflammatory response in day and night (Figure 2C & D). Such disruption is closely associated with the abolishment of the circadian rhythms of circulating insulin, glucose and lipid [14,15] and needs correction. Taken together, overweight and obese patients have an increased risk of developing Type 2 diabetes, owing to disruption of the circadian glucose-lipid control between nightly catabolic-mediated lipid utilization and daily anabolic-mediated glucose expenditure and/or storage. Such circadian abnormalities serve as a pathological metabolic basis for prediabetes, because of their potential ability to elevate the IR threshold, in parallel with the increased insulin and FFA levels in day and night periods without glucose growth. Long duration of these circadian disorders can result in a shift to diabetes by creating a vicious cycle between inflammatory-induced growing FFA (as in day and night periods) and insufficient increment of insulin production, which cannot compensate for the increased IR-threshold and tends to elevate the glucose level in blood. What strategy might be used to counter these circadian metabolic disorders?

## Catabolic shift in prediabetic state as target for therapeutic correction

One of important implications of the above mentioned ana­lysis is that transition of an adaptive IR to its chronic pathological state is followed by the activation of catabolic programs (as lipolysis and low-grade inflammation) under anabolic conditions, which is associated with elevated circulating insulin concentration in day and night period. In consequence of these changes, chronic IR may disrupt the circadian rhythm that promotes the development of the obesity-induced prediabetic state. For example, permanent IR supports uncontrolled lipolysis, which is one of the earlier events in systemic lipotoxicity [26,28]; thus insulin-sensitizing therapies with as short-term treatment with pioglitazone can help increase lipid storage in the adipose tissue to overcome lipotoxic effects [29,30]. The finding that adipose triglyceride lipase deficient mice are insulin sensitive despite triglyceride accumulation suggests that lipase can also be targeted for metabolic disease treatment [29]. Experimental verification of this suggestion demonstrated that the use of different inhibitors of adipose tissue lipolysis may improve insulin sensitivity and glucose metabolism [31,32]. It is important to note that the widely used clinical antidiabetes drug metformin can also suppress the lipolytic response to catecholamine or TNF-α [33,34]. This result confirms that metformin is capable of decreasing plasma FFA-levels and improving insulin sensitivity in parallel with hindering hepatic glucose production from lipids [35]. Metformin and resveratrol also ameliorate muscle IR through preventing lipolysis and inflammation in the hypoxic adipose tissue [17,18] by blocking the FFA trafficking under conditions of adipose dysfunction. Magnesium supplementation can also improve insulin sensitivity even in normo-magnesemic, overweight subjects emphasizing the need for an early optimization of magnesium status to prevent IR and subsequently Type 2 diabetes [36]. Rutin treatment also significantly reduced adiposity and improved glucose homeostasis in both genetically and diet-induced obesity mice [37].

Before formulating therapeutic strategies based on circadian correction, certain questions remain to be addressed. Can therapeutics be developed that specifically target the chronic inflammation typical for prediabetic state? It was found that some anti-inflammatory drugs are capable of reducing the progression of diabetes, namely salicylates and aspirin, because they can inhibit the NF-kB pathway via its ability to improve glucose metabolism and diabetes [38,39]. Amelioration of the inflammatory response and IR is observed following inhibition of the CD8^+^ T-cell action and activation of Treg cell action that are produced in large amounts of the anti-inflammatory cytokine interleulin-10, which itself has insulin sensitizing effects [40]. It is important to note that there are certain diet nutrients with anti-inflammatory potential, such as lycopene, vitamin C and resveratrol, which can improve the obesity-associated metabolic-inflammation [41] but for which effects on circadian metabolic disorders are uninvestigated.

Another possible way to counter the circadian metabolic disruption is the use of clock-driven hormones (melatonin), which can act as an entrainment signal for the circadian system. However, both inhibitory and stimulatory effects of melatonin on insulin secretion and glucose metabolism have been reported *in vivo* and *in vitro* [42,43]. It was found that melatonin has a significant negative effect on glucose clearance in the morning, but not at night [42]. Moreover, melatonin treatment inhibits insulin secretion, which increases the risk of higher glucose levels, as increased melatonin signaling was proposed as a risk factor for Type 2 diabetes [43]. However, it is unclear why, in a large clinical trial, exploring the use of melatonin receptor agonists as therapeutic agents for Type 2 diabetes grossly impaired glucose tolerance and perhaps even obesity [43]. Therefore, further investigation is required.

The other strategy of a potential decline of prediabetic symptoms is the decrease of blood glucose level upon application of insulin treatment as a glucose-lowering drug. Insulin is widely used for this aim in modern clinical practice but many questions remain to be answered about the negative side effect of insulin action. One of them is diabetes relapse in patients with growing weight due to insulin-stimulated fat accumulation [44]. This clinical observation complies well with the model that is presented in the graphic abstract. The model predicted that some doses of insulin are able to stimulate the fast glucose uptake and its accumulation as fat in hypertrophic adipocytes that may trigger the circadian cycle, leading to relapse of diabetes. Moreover, intensive insulin therapy in overweight or obese patients can lead to a dramatic increase in myocardial triglyceride content with a lipotoxic effect, whereas the induction of IR in heart can be considered as a protective response [45,46]. In addition to this fact, an increased insulin level inhibits cardiac contractility and inhibits sodium excretion by increasing sodium re-absorption in the kidney, which increases the risk to hypertension [47]. In the final ana­lysis, Nolan *et al*. [45] and Taegtmeyer *et al*. [46] come to the unexpected conclusion that the anabolic-associated IR is a defense mechanism that protects critical tissues of the cardiovascular system from over-nutrient-induced injury. Overriding IR in an effort to lower plasma glucose levels, particularly with intensive insulin therapy, could therefore be harmful [47,48]. Moreover long-term hyperinsulinemia as in night and day period [14] is the platform for retention of obesity (Figure 2C & D) with low-grade inflammation. Therefore, approaches to the managements of these overweight patients should include reduction in excess fuel supply and nutrient off-loading in parallel with the increased physical activity.

Taken together, there are various therapeutic strategies that could target diabetes or prevent its development. Prediabetes may be evolved by circadian disorders in glucose and lipid metabolism that is a result of the catabolic activation, namely chronic inflammation and lipolysis, in anabolic conditions typical of overweight and obesity. The present ana­lysis supports the suggestion that inhibition of low-grade inflammation/lypolysis can reduce the risk for development of diabetes.

## Conclusion

IR is nearly always considered to be ‘harmful’ and at the root of Type 2 diabetes [49]. However, in biological evolution the negative regulation of insulin sensitivity could be viewed as an essential part of the adaptive two-sided mechanism acting under opposite catabolic and anabolic conditions [4]. The main aim of this physiological IR is the host survival through maintenance of glucose homeostasis under critical time of low/high nutrient availability as in the circadian clock or high energy needs and ability to fight infection or stress/trauma [11]. During the circadian period of food restriction, protection against hypoglycemia is accomplished by reduction of insulin secretion and activation of a catabolic program. It could be lipolysis of adipocytes, leading to release of lipids and FFA for energy use and hepatic glucose production in parallel with blocking glucose delivery into muscle and fat cells. The latter can be named catabolic IR and its consequence is redirection of glucose to other high glucose-dependent tissues such as the brain. In contrast when nutrients are plentiful, anabolic signaling ensures insulin-stimulated glucose uptake in adipocytes for energy storage. This pathway is controlled via activation of the anabolic IR response, especially in fat tissue restricting its hypertrophic state. Professor Straub noted that in early human evolution catabolic IR has a peak incidence [50], in contrast to present lifestyle with low physical activity and great food availability that leads to overweight and anabolic IR response. To explain the above facts in the adipocytes circadian system, the U-type dose-response model of two distinct actions of IR and its relationship with the threshold of IR, blood glucose concentration, activation of anabolic/catabolic programs is proposed (Figure 3). It is known that insulin sensitivity in healthy human adipose tissue shows an endogenous circadian rhythm, with reduced sensitivity only at night in parallel with activation of lipolysis [9]. This rhythm can be impaired in overweight and obesity patients wherein IR becomes a chronic response and gains an elevated threshold to insulin action. This pathological form of IR can be considered as a result of chronic day–night activation of catabolic pathways (low-grade inflammation and lipolysis) in hypertrophic adipocytes tissue, which have no more reserves for fat accumulation. Prolonged maintenance of this conflict metabolic state can form the pathological basis for circadian disruption, which development came before the development of obesity-induced diabetes (Figure 3, indicated with red color). The cancer-induced metabolic situation supports catabolic IR [3] that is just opposite anabolic IR typical of overweight and obesity (Figure 3, indicated with blue color). The proposed U-like curve model illustrates the principle when transient adaptive IR become chronic and which expands our understanding for targets of its correction. However many questions remain unanswered, such as the roles of hepatic lipids in hepatic IR and Type 2 diabetes [51].

Taken together, the analyzed data support the idea that the IR acts as a dual complementary adaptive mechanism for maintenance of glucose homeostasis in opposite catabolic and anabolic conditions. Under these conditions catabolic IR can protect brain tissues from hypoglycemic shock, whereas anabolic IR can protect critical tissues of cardiovascular system from hyperglycemia-induced injury. This mechanism may provide the basis for a new approach to reduce the risk for induction of prediabetes state and to develop a new therapy for normalization of metabolism in overweight humans.

## Future perspective

In recent years, the important role of the circadian system in the control of glucose metabolism has gained clinical interest based on epidemiological data linking western lifestyles related to circadian disorders to increased risk of obesity and Type 2 diabetes. A recent review [43] assembled the different circadian mechanisms and separate metabolic signal-regulators involved in diabetes development. How such separate signals are integrated into a coordinated circadian response in healthy (not in obese) individuals remains without answer and is a crucial area of investigation. In the present article a new integrated hypothesis is developed that is capable of answering this question and proposes its common circadian mechanism, in which only two main metabolic programs (lipid catabolism and its anabolism) are considered along with their primary action on opposite circadian periods. Circadian switching between these programs is associated with the reciprocal activation of glucose, insulin and lipid metabolism. However such switching in overweight and obese patients is impaired, in parallel with increment of the IR-threshold by hyperinsulinemia and induction of low-grade inflammation. The simple feedback model (Figure 2) of this circadian disturbance may help in active search of a new therapeutic treatment for prevention or reversion of prediabetes. According to this aim, the results derived from the existing viewpoint should help to determine the unexpected negative consequences from glucose-lowering treatment with insulin, which is attended with increased levels of anabolic fat accumulation and formation of hypertrophic adipocytes. In turn, these hypertrophic adipocytes release elevated amounts of FFA and proinflammatory cytokines in day/night periods. This pathological state is an increased risk for repeated development of obesity-associated diabetes in part by inducing increment threshold of IR. A simple model of insulin-driven vicious cycle in the hypertrophic adipocytes is presented in this paper's ‘Graphical abstract’. This negative side effect of insulin treatment must be investigated for detailed elaboration of clinical recommendations in future.

Viewing diabetes as a metabolic circadian disease with anabolic/catabolic imbalance, it can be proposed that certain diet and/or physical exercise can restore the healthy circadian rhythm via reduction of IR, FFA and blood glucose levels. As an example, this aim may be achieved by exercise through trained skeletal muscles, because intensive exercise increases consumption of glucose by up to 50-fold and in this way improve hyperglycemia and also counteract IR [52]. Although the combination of a low-caloric diet and regular physical exercises leads to weight loss, the more stable result can be achieved by applying the anti-obesogenic and antidiabetic plants, which reduces energy storage via inhibition of anabolic pathways and enhances energy expenditure via stimulation of lipid catabolism [53]. This positive result can be also improved by reduction of acetate production in gut microbiota that is able to reduce obesity in animals early receiving the high-fat diet. Therefore, the changes in gut microbiota are another candidate for reversion of circadian IR, obesity and diabetes [54]. Future studies are needed to check these perspective research directions with the aim to provide insight into the metabolic disease initiation and progression that impact its detection, prevention and therapy.

**Executive summary**

In recent years, the role of the circadian system in the control of glucose-lipid metabolism gained clinical interest based on epidemiological data linking lifestyles related to circadian disorders to increased risk of obesity and type 2 diabetes that are associated with chronic insulin resistance in day and night period. Therefore, the goals of this study were: to investigate the relationship between anabolic/catabolic insulin resistance and circadian metabolism via comparison of reciprocal glucose–lipid regulation in lean, overweight, obese and diabetic individuals; to test and determine whether obesity-increased insulin and free fatty acid levels are sufficient the cause of chronic insulin resistance in day–night period as a pathological basis for diabetes development; to devise a simple feedback model of the key metabolic circadian disorders leading to chronic insulin resistance and diabetes; to analysis whether a lipid catabolic shifts in anabolic conditions typical for obesity and pro-diabetic state can be a target for therapeutic correction.
